# Genome Sequences of 11 Shiga Toxin-Producing *Escherichia coli* Strains

**DOI:** 10.1128/genomeA.00418-15

**Published:** 2015-05-14

**Authors:** C. Gabrielsen, F. Drabløs, J. E. Afset

**Affiliations:** a Department of Laboratory Medicine, Children’s and Women’s Health, Faculty of Medicine, Norwegian University of Science and Technology, Trondheim, Norway; b Department of Cancer Research and Molecular Medicine, Faculty of Medicine, Norwegian University of Science and Technology, Trondheim, Norway; c Department of Medical Microbiology, St. Olavs University Hospital, Trondheim, Norway

## Abstract

Shiga toxin-producing *Escherichia coli* (STEC) strains are a common cause of both sporadic infection and outbreaks of enteric disease in humans. Here, we present draft genome sequences of 11 STEC strains of different serotypes (O145, O121, O26, O177, and O-type unknown), that have been isolated from patients with enteric disease of various degrees of severity, in the years 2001 to 2014 at St. Olavs Hospital in Trondheim, Norway.

## GENOME ANNOUNCEMENT

Shiga toxin-producing *Escherichia coli* (STEC) strains are a common cause of both sporadic infections and outbreaks of enteric disease in humans, the symptoms of which range from asymptomatic carriage to hemolytic uremic syndrome (HUS). The main virulence factor of these bacteria is the Shiga toxin, which acts to inhibit protein synthesis in target cells, as well as the locus of enterocyte effacement (LEE) pathogenicity island, the latter being important for adherence to host intestinal epithelial cells. In order to study the genetic content of STEC in relation to its degree of virulence, we sequenced 11 STEC strains that have been clinically characterized at St. Olavs Hospital, Trondheim, isolated from patients with known severity of enteric disease, containing Shiga toxin type 2a and the LEE pathogenicity island.

Strains were grown overnight on MacConkey agar, and genomic DNA was isolated using the Qiagen MagAttract DNA Mini M48 kit and the Qiagen BioRobot M48 workstation (Qiagen, Hilden, Germany) as described by the manufacturer.

Libraries were prepared using the TruSeq Nano DNA sample prep kit (Illumina, San Diego, CA, USA), and the libraries were sequenced on the Illumina MiSeq platform (Illumina) with 300-bp paired-end read configuration. The sequencing service was provided by the Norwegian Sequencing Centre (http://www.sequencing.uio.no).

Raw read quality was assessed using FastQC (1), and low-quality reads were trimmed using Trimmomatic (2). Overlapping reads were merged with FLASH (3). Paired-end and orphan reads were then *de novo* assembled using SPAdes Genome Assembler version 3.5.0 (4), with a minimal coverage cutoff of 20 and a minimum length of 500 bp. QUAST version 2.3 (5) and REAPR version 1.0.17 (6) were used for assessing the quality of assemblies. Assemblies were subsequently annotated using NCBI’s Prokaryotic Genomes Annotation Pipeline.

The average genome size of the sequenced strains is 5.5 Mbp, while the average scaffold number is 208 and the GC content is 50.5%. The sequenced strains encode on average 5,507 protein-coding genes, 17 rRNA genes, and 92 tRNA genes, and as is commonly observed in STEC, the strains contained high numbers of bacteriophage- and mobile element–related sequences producing large numbers of small contigs.

This study was approved by the Regional Committee for Medical and Health Research Ethics, REC, South-East (REC number 2011/2314).

### Nucleotide sequence accession numbers. 

The draft genome sequences for these 11 Shiga toxin-producing *E. coli* strains have been deposited at DDBJ/EMBL/GenBank under the accession numbers listed in Table 1. The versions described in this paper are the first versions.

**TABLE 1:** GenBank accession numbers of sequenced STEC strains

Strain	Accession no.
St. Olav17	JYKT00000000
St. Olav39	JZDU00000000
St. Olav40	JZDV00000000
St. Olav63	JZDW00000000
St. Olav157	JZDX00000000
St. Olav172	JZDY00000000
St. Olav173	JZDZ00000000
St. Olav174	JZEA00000000
St. Olav176	JZEB00000000
St. Olav178	JZEC00000000
St. Olav179	JZED00000000
